# Factors associated with program effectiveness in the implementation of a sexual risk reduction intervention for female sex workers across Mexico: Results from a randomized trial

**DOI:** 10.1371/journal.pone.0201954

**Published:** 2018-09-11

**Authors:** Eileen V. Pitpitan, Shirley J. Semple, Gregory A. Aarons, Lawrence A. Palinkas, Claudia V. Chavarin, Doroteo V. Mendoza, Carlos Magis-Rodriguez, Hugo Staines, Thomas L. Patterson

**Affiliations:** 1 Department of Medicine, University of California San Diego, La Jolla, California, United States of America; 2 Department of Psychiatry, University of California San Diego, La Jolla, California, United States of America; 3 School of Social Work, University of Southern California, Los Angeles, California, United States of America; 4 Evaluation and Research Department, Mexican Foundation for Family Planning (Mexfam), Mexico City, Mexico; 5 Centro Nacional para la Prevencíon y Control del VIH/SIDA (CENSIDA), Mexico City, Mexico; 6 Universidad Autonoma de Ciudad Juarez, Ciudad Juarez, Chihuahua, Mexico; TNO, NETHERLANDS

## Abstract

**Objective:**

The overall aim of this paper is to examine effectiveness of an evidence-based intervention in community settings, and the factors associated with effectiveness. Limited research in the area of HIV prevention has focused on evaluating intervention program effectiveness in real-world settings.

**Methods:**

We implemented an efficacious theory-based sexual risk reduction intervention for female sex workers (FSW) called *Mujer Segura* across 13 different clinics in 13 sites across Mexico. The overall design was a cluster randomized Type I design simultaneously testing intervention program effectiveness with an observational study of implementation factors. We aimed to examine the effectiveness of *Mujer Segura* at reducing HIV/STI incidence among FSW participants at each site, and to examine the client-, provider-, organization-, and structure-related factors associated with program effectiveness.

**Results:**

We found lower HIV/STI incidence density in the intervention relative to the control group in 5 sites we labeled as “program effective sites,” but not in 8 sites we labeled as “program ineffective sites.” Using generalized estimating equations controlling for site and computed mean difference effect sizes, we examined statistically and practically significant differences, respectively, between the two groups of sites along various client-, provider-, organization-, and structure-related characteristics. Results indicated that client-level HIV/AIDS related knowledge, and proficiency and engagement in the organizational social context were associated with program effectiveness.

**Conclusions:**

Enormous resources are required to systematically and adequately test the role of multilevel factors on program effectiveness. We successfully implemented *Mujer Segura* in 13 sites in Mexico. Results suggest that other measures may need to be included in future implementation studies than the ones included here. We were able to point to a few specific factors that should be targeted to increase effectiveness of similar evidence-based programs in low- and other middle-income countries like Mexico.

**Trial registration:**

ClinicalTrials.gov NCT01465607.

## Introduction

Female sex workers (FSW) in Mexico have an elevated HIV prevalence compared to the general population [1]. In a study of FSW conducted along the US-Mexico border, HIV prevalence was estimated at 6% compared to 1% in the general population [1]. High rates of condomless vaginal and anal sex as well as low rates of condom negotiation with clients have been reported among FSW in this low-to-middle income country (LMIC) [2,3]. In 2007 our research team demonstrated the efficacy of a brief (35-minute) theory-based behavioral intervention called “*Mujer Segura*” (“*Safe Woman*”) to promote condom use and enhance safer sex negotiation skills and reduce HIV/STI risk among FSW in two U.S.-Mexico border cities. In a randomized controlled trial, we found that FSW randomized into *Mujer Segura* had a 40% lower cumulative incidence of sexually transmitted infections [2]. There were also concomitant increases in total numbers and percentages of protected sex acts and decreases in total numbers of unprotected sex acts with clients (p<0.05) at six-month follow-up. In collaboration with Mexican government officials, and the Mexican Foundation for Family Planning (Mexfam), a non-governmental organization focused on women’s reproductive health, we conducted a large-scale implementation and effectiveness trial of *Mujer Segura*. Mexfam has clinic sites throughout Mexico, and we implemented the intervention program across 13 different Mexfam community clinics in 13 sites across Mexico. The general aim of the current paper is to examine the effectiveness of the intervention in the context of real-world community settings.

Despite a preponderance of studies in the HIV prevention literature that aim to develop and test the efficacy of behavioral interventions, less is known about whether efficacious interventions also demonstrate effectiveness in less controlled, real-world community settings. Even less is known about the factors in real-world community-based settings that may or may not affect effectiveness of evidence-based programs. The effectiveness of evidence-based programs within community settings may be affected by factors from different sources that occur within multiple system and organizational levels [4]. The implementation science literature has identified the types of factors that are hypothesized to affect implementation–and ultimately, the effectiveness–of evidence-based health innovations, which can be organized into client-, provider-, organization-, and structural-related factors [4–7].

Client-related factors include demographic characteristics (e.g., gender), behavioral risk factors (e.g., substance use), as well as knowledge, beliefs, self-efficacy, and motivation to change behavior [8–11]. Although client-level variables are examined most often in efficacy trials, there is evidence to suggest that these factors also have an important influence on the implementation process [5]. Provider factors can also significantly affect the effectiveness of evidence-based interventions [12–14], including: attitudes toward adoption of innovation (e.g., intuitive appeal of the evidence-based program) [15]; personal dispositional innovativeness (e.g., adaptability); and knowledge and perceived utility of the evidence-based program [15–17]. Leadership and the organization’s social context (i.e., culture and climate) are organization-related factors that are associated with more positive provider attitudes toward evidence-based programs [12,18]. Structural or community-level factors are also potentially important to the understanding of implementation and program effectiveness. These factors include the political and social climate (e.g., support for funding), economic climate (e.g., availability of public versus private funding), and infrastructure in the community (e.g., availability of public transportation). To date, few studies have examined the effects of structural factors on implementation processes or program effectiveness [6,19].

Implementation frameworks and models suggest that the achievement of effectiveness in evidence-based programs are the natural result of a successful implementation process. There often remains a gap of knowledge in the transition from efficacy in controlled trials to effectiveness in real-world settings [20]. Instead, effectiveness is often assumed after demonstrating efficacy in efficacy trials. The current study has produced published data on implementation-related outcomes, including fidelity [21], sustainability [22], and feasibility and acceptability [23]. In the current paper, the outcome we focus on is the *effectiveness* of the intervention. We examined the effectiveness of *Mujer Segura* in each clinic (i.e., whether the intervention significantly reduced HIV/STI incidence and reduced sexual risk behaviors), and examined the client-, provider-, organization-, and structure-related characteristics that might explain differences in program effectiveness. We hypothesize that provider- and organization-related factors known to be associated with successful implementation will also be associated with effectiveness in this study. These include more positive evidence-based practice attitudes or a more positive organizational social context (e.g., less rigidity and stress, more proficiency and engagement), and higher emotional competence. Client- and structural- related factors have been studied less in the area of effectiveness; therefore we aim to explore how these factors might be related to effectiveness in this study.

Our approach to studying factors related to program effectiveness focuses on characteristics that operate at multiple levels (i.e., client, provider, organization, and broader structure). However, we did not conduct multilevel modeling to analyze the data. Instead, while we still controlled for the nested structure of the data, we elected a more parsimonious approach and divided sites into those that were “program effective” (i.e., the intervention program was effective at reducing STI incidence and reducing condomless sex at the site) and “program ineffective,” and compared the two groups along different multi-layered characteristics.

## Method

### Setting

This study took place in 13 clinics in eight states in Mexico. Clinics were located in Veracruz, Iguala, Ixtaltepec, Revolución, Nezahualcoyotl. Naranjos, Tuctla, San Luis Potosí, San Luis de la Paz, Tepeji del Río, Guadalajara, Huajuapan, and Tlapa. The clinics primarily served women in regard to family planning, reproductive health, and childbirth.

### Overview of design

**Fig 1:**
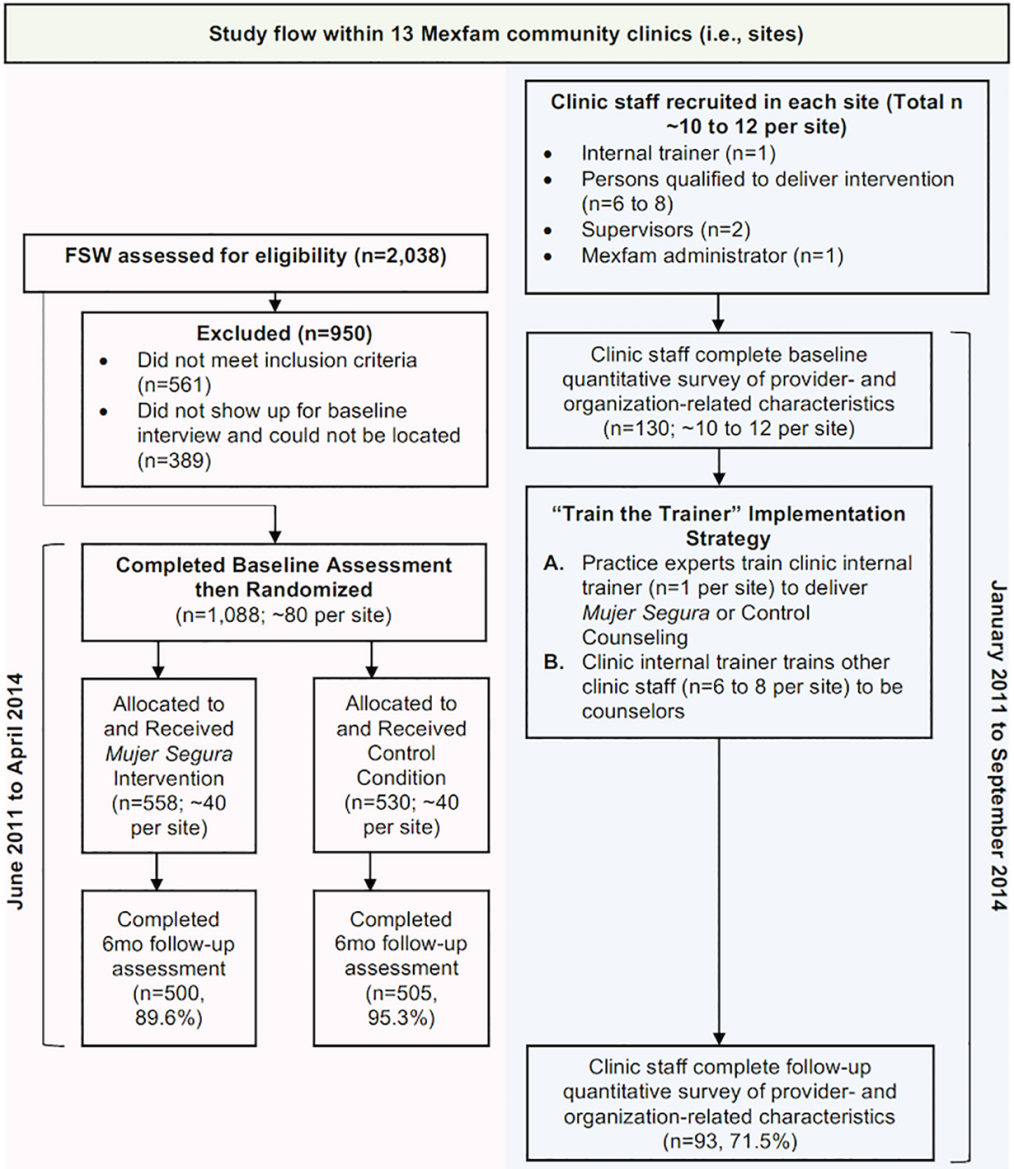
Study flow of cluster randomized Type I design simultaneously testing intervention program effectiveness (pink) with an observational study of implementation factors (blue) across 13 clinics in Mexico.

The overall design of the study was a cluster randomized (participants randomized within clinic) Type I design that simultaneously tested intervention program effectiveness with an observational study of implementation factors [24]. Details of the methods and power calculations are described in the study protocol by Patterson et al. [25]. This study involved two parallel procedures which are summarized in Fig 1. The first involved a multi-site, randomized controlled trial (RCT), with two arms and a 50/50 allocation ratio to either *Mujer Segura*, or a time-equivalent attention control condition. Thirteen different clinics in eight states in Mexico were enrolled and FSW were randomized within clinics with a target of recruiting 80 FSW per clinic. The selection process of the clinics is described in the protocol; generally, sites that had the capacity to implement the intervention were selected. We originally aimed to only include 12 clinics, but added a thirteenth after the study protocol was published because the first study site (Revolución) was slow to start due to drug-related violence in the area. The second aspect of the study, which was not randomized, involved the collection of data at the clinic sites concerning provider, organizational, and structural factors hypothesized to affect the effectiveness of the intervention. The implementation strategy was a “train the trainer” approach that was designed to build a network of HIV prevention services for FSW that would achieve sustained fidelity and provider competency [22].

### Participants and procedures

#### Female sex workers

Eligibility criteria for the FSW were the same as those of the previous efficacy study. Participants were biologically female; at least 18 years of age; self-identifed as a FSW; report having traded sex for drugs, money, shelter, or other material benefit within the previous two months; had unprotected vaginal or anal sex with a client at least once during the previous two months; had no previous HIV-positive test result; and agreed to be tested for HIV and STIs at baseline and at 6-month follow-up. Female sex worker participants completed a baseline assessment, were randomized into *Mujer Segura* or the control group using a computer-generated random order, and completed a 6-month follow-up assessment. In total, 1,088 FSW participated in this aspect of the study. A total of 558 were randomized into the experimental condition and 530 into the comparison condition. Staff at each site enrolled participants and assigned participants to conditions. Baseline and follow-up procedures were completed between June 8, 2011 to April 30, 2014.

Those in the experimental condition received the *Mujer Segura* intervention, a brief (35 to 40 minute), single-session, individual intervention that combines principles of motivational interviewing (MI) [9], social cognitive theory (SCT) [8], and the theory of reasoned action [26]. A detailed description is provided elsewhere [27]. The counselor uses motivational interviewing techniques (e.g., key questions, reflective listening, summarization, affirmation, and appropriate use of cultural cues) to increase the participant’s motivations to practice safer sex. Those in the control condition received standardized counseling that was equivalent in time and attention.

Because FSW are at high risk of STI and HIV infection, both interventions impart knowledge necessary for practicing safer sex. However, in the standard counseling condition, topics are covered in a lecture format without the use of MI or the role-plays, exercises, and problem-solving emphasized in the *Mujer Segura* intervention. The comparison condition is time-equivalent and utilizes counseling materials provided by CENSIDA, the Mexican federal agency responsible for HIV and AIDS prevention [28], that are currently in use at the clinics.

#### Clinic personnel

Across the 13 sites a total of 130 clinic staff completed a baseline battery of measures related to attitudes towards evidence-based practice, and organization attitudes and practices. Data were collected in-person, on-site at each clinic between January 11, 2011 to December 6, 2013. These measures include emotional competence, leadership, and organizational social context.

### Ethics statement

All study procedures were approved by the Institutional Review Board of the University of California, San Diego (Project 090320) and by the Ethics Committee at Mexfam. All procedures were conducted in accordance with the Helsinki Declaration. Written informed consent was obtained from all participants.

### Measures

#### Primary and secondary outcomes for program effectiveness

To evaluate program effectiveness, our primary outcome was the 6-months incidence density of HIV/any STI by site, calculated using person-time methods. More specifically, the incidence density was calculated by dividing the number of incident cases that occurred during the follow-up by the number of person time (years) spent at risk. For the women who did not acquire HIV or an STI during the follow-up, the time at risk was represented by the entire time period between the baseline- and their 6-month visit, whereas, for the women who acquired either HIV or an STI, the time at risk was represented by half of the time interval between the baseline and their 6-month visit.

Our secondary outcome was behavioral, the number of unprotected sex acts with clients during the previous 30 days (reported at 6 months).

#### Client-related factors

FSW participants answered questions about their age, marital status, and age when they first started trading sex. We also asked about their alcohol use using the AUDIT-C scale; a three-item, reliable alcohol screening instrument that measures frequency and quantity of alcohol consumption using a 5-point scale for each item. Summary scores were used to create a dichotomous variable (hazardous versus non-hazardous drinking). Based on validation studies, a score of 3 or more for women was used to identify risky or hazardous drinking [29, 30]. Participants also reported whether they used heroin, cocaine, and/or methamphetamine in the past month (in separate items). We also asked about how often they used alcohol or an illegal drug (in separate items) before or during sex with a client (1 = never, 2 = sometimes, 3 = often, 4 = always). They also provided ratings of their overall financial situation (1 = extremely good to 5 = extremely bad) and overall working conditions (1 = extremely good to 5 = extremely bad). Finally, they answered questions about their sexual behaviors with clients: total number of clients, and total number of unprotected vaginal and anal sex acts, both in the past month.

We assessed HIV-related knowledge using the 18-item HIV knowledge questionnaire [31], with higher scores indicating higher knowledge. Using items we developed in our previous studies with FSW in Mexico [27], we also assessed condom use self-efficacy, condom use outcome expectancies, and attitudes towards condoms. Condom use self-efficacy was measured with four items, including “I can use a condom every time I have vaginal or anal sex.” Response options ranged from 1 = strongly disagree to 4 = strongly agree. Condom use outcome expectancies was measured with five items, including “I believe that condoms interfere with sexual pleasures;” and “My client will not be sexually satisfied if we use a condom.” Higher scores indicate more negative outcome expectancies for condom use. Response options ranged from 1 = strongly disagree to 4 = strongly agree. Attitudes towards condoms was measured with three items, including “My always using a condom during vaginal intercourse with my clients during the next month would be…” Response options ranged from 1 = very bad to 3 = neither good nor bad to 5 = very good.

#### Provider-related factors

Personnel from each site completed measures of organizational and provider factors. Specifically, they completed measures of evidence-based practice attitudes and emotional competence. For these two measures, we used the same response scale (0 = not at all to 4 = to a very great extent). We also assessed counselor fidelity to the intervention as a provider-related factor.

**Evidence-based practice attitudes.** Personnel completed a measure of their attitudes toward the adoption of evidence-based practices using the Evidence-Based Practice Attitude Scale [15,32]. This is a 15-item scale with four subscales measuring intuitive appeal (4 items, example item: “If you received training in a therapy or intervention that was new to you, how likely would you be to adopt if it ‘made sense’ to you,” α = 0.86), attitudes toward organizational requirements (3 items, example item: “If you received training in a therapy or intervention that was new to you, how likely would you be to adopt if it was required by your agency,” α = 0.93), openness to innovation (4 items, example item: “I am willing to use new and different types of therapy/interventions developed by researchers,” α = 0.87), and perceived divergence of research-based innovation (4 items, example item: “Research based treatments/interventions are not clinically useful,” α = 0.60).

**Emotional competence.** Emotional competence is the learned capability based on emotional intelligence, or knowing and handling one’s own and others’ emotions that results in outstanding performance at work [33]. The Emotional Competence Inventory is designed to capture emotional intelligence through the “competencies that constitute self-awareness, self-management, social awareness, and social skills at appropriate times and ways in sufficient frequency to be effective in a given situation” [33,34]. The inventory consists of four clusters: self-awareness including emotional self-awareness, accurate self-assessment, and self-confidence (16 items, α = 0.88); self-management including self-control, conscientiousness, and adaptability (30 items, α = 0.93); social awareness including empathy, organizational awareness, and service orientation (17 items, α = 0.92); and social skills including leadership, communication, influence, conflict management, and teamwork and collaboration (46 items, α = 0.97).

**Intervention fidelity.** FSW participants who were randomized into the experimental arm and completed the *Mujer Segura* intervention were asked to complete a checklist of all of the intervention tasks and elements that were or were not completed by their counselor. There was a total of 40 intervention tasks that each *Mujer Segura* counselor was to complete. We created a dichotomous fidelity variable of whether the participant reported that the counselor completed all intervention tasks. Participants in the control condition did not complete a measure of fidelity.

#### Organization-related characteristics

Personnel from each site completed measures of evidence-based practice staffing and recognition, transformational leadership, and organizational social context. For these, we used the same response scale as the one used for the provider-related measures (0 = not at all to 4 = to a very great extent).

**Evidence-based practice staffing.** Participants indicated their level of agreement with different items about whether the clinic selects staff that would support evidence-based programs [15]. There are 8 items in this measure (α = 0.92), with example items being “This clinic selects staff open to new types of interventions;” and “This clinic selects staff who are flexible”.

**Evidence-based practice recognition.** Participants indicated their level of agreement with different items about whether the clinicians who use interventions would receive positive recognition [15]. There are 8 items in this measure (α = 0.89), with example items being “Clinicians who use interventions based on scientific publications are held in high esteem in this clinic,” and “In this clinic, the more you know about interventions based on scientific publications, the better your chances are of getting promoted”.

**Transformational leadership.** The Multifactor Leadership Questionnaire [35] was used to assess transformational leadership. Participants were asked to answer questions in regard to their own supervisor at the clinic. Transformational leadership includes four subscales: individualized consideration (4 items, example item: “Treats you as an individual rather than just a member of the group,” α = 0.82), intellection stimulation (4 items, example item: “Seeks differing perspectives when solving problems,” α = 0.82), inspirational motivation (4 items, example item: “Expresses confidence that goals will be achieved,” α = 0.89), and idealized influence (8 items, example item: “Specifies the importance of having a strong sense of purpose,” α = 0.82).

**Organizational social context.** The organizational social context (OSC) measure includes eight subscales designed to capture organizational culture, climate, and employee morale [36]. Participants reported in regard to their own clinic. Culture refers to behavioral norms and expectations that govern how things are done in an organization and includes the dimensions of rigidity–or the extent to which clinicians have little input over and flexibility in carrying out their jobs (13 items, α = 0.73); proficiency–or the extent to which clinicians place the well-being of clients first, as well as competency and knowledge (15 items, α = 0.93); and resistance–or the extent to which clinicians show little interest in change of providing service (12 items, α = 0.70). Climate refers to organizational climate and includes the dimensions of engagement–or the extent to which clinicians perceive they are able to accomplish tasks and be personally involved in their work and have concern about clients (10 items, α = 0.65); functionality–or the extent to which clinicians perceive cooperation and help from coworkers and administrators, and a clear understanding of their roles within the organization (15 items, α = 0.86); and stress–or the extent to which clinicians are emotionally exhausted and overloaded from work (20 items, α = 0.83). Morale is an individual-level construct and refers to the extent to which the clinicians are committed to the organization and are satisfied with their job (17 items, α = 0.92).

#### Structure-related characteristics

The marginalization index was computed for each city in Mexico in 2010 by the Consejo Nacional de Población [37]. A higher marginalization index indicates that the “sector of society is in a situation in which they are not presented opportunities for development, nor do they have the ability to find such opportunities” [37]. The index has been used in previous HIV/AIDS research in Mexico [38]. The United Nations Human Development Index is comprised of health, education, and income indicators [39] and is available for each Mexican state [40]. We gathered this data in 2014 at the conclusion of the study, which overlaps with the time period of data collection for the intervention outcomes (i.e., STI incidence, sexual risk behavior). We examined each indicator separately as predictors. The health index is calculated from life expectancy at birth, education from mean years of schooling and expected years of schooling, and income from the gross national income per capita.

### Analysis plan

We analyzed the data in steps. In the first step, for both the intervention and control groups we calculated our primary outcome, 6-months incidence density of HIV/any STI by site, using person-time methods. The thirteen sites were then individually categorized as a “program effective” or a “program ineffective” site, with a “program effective” site being defined as a site that yielded a lower incidence density in the intervention group as compared to the control group. The two site groups were then compared on client-, provider-, organization-, and structural-related characteristics by level of success. We used different data sources depending on the type of characteristic in question. For client-related characteristics, we compared FSW participants from program effective versus program ineffective sites (n = 1,088) on their baseline characteristics. For provider-related characteristics, we compared clinic personnel from program effective versus program ineffective sites (n = 130) on their responses to provider-related questions. This was the case for all provider-related measures except intervention fidelity. Since the latter was completed by the FSW participants, we compared a total of 558 women who completed the *Mujer Segura* intervention from the program effective and program ineffective sites. For organization-related characteristics, we also compared responses from clinic personnel from program effective versus program ineffective sites (n = 130) on their responses to questions about their organization/clinic. For structure-related characteristics, we compared the 13 total program effective and program ineffective sites on population total, and health, education, income, and marginalization indices.

We aggregated the different sources of data differently depending on the level/type of characteristic in question (i.e., client, provider, organization, and structure). For client-related characteristics, we aggregated data from female sex workers (n = 1,088). For provider and organization characteristics, we aggregated data from clinic staff across the 13 sites (n = 130). For structure-related characteristics, we analyzed each clinic as its own unit (n = 13). Regardless of the type of characteristic, we divided data from program effective sites and compared them with data from program ineffective sites (e.g., FSW and clinic staff from those sites). To compare sites, we examined both statistical (i.e., p-values) and practical (i.e., effect sizes) significance. To analyze statistically significant differences between program effective and program ineffective sites, we modeled the data using generalized estimating equations (GEE). GEE allows us to adjust for site clustering, and appropriately model the distribution of the outcome. We conducted GEE to predict whether the particular unit of analysis (e.g., FSW or clinic staff) was part of a program effective or program ineffective site using a binomial distribution and logit link function. In the GEE models, we specified an exchangeable covariance structure. P-values from GEE model results are presented in the tables. In addition to examining statistically significant differences, we also computed effect sizes for differences between program effective and program ineffective sites. Effect sizes shed light on practical or clinical significance, rather than statistical significance. They also help with the analyses of the structure-related variables specifically, since we have an analytic sample of only 13 for these variables [34].

We followed the procedures described by Lipsey and Wilson to calculate and interpret the magnitude of effect sizes [41,42]. For each variable, we used different calculations and effect size measures depending on the type of predictor variable (continuous or dichotomous). The effect size measures we used were the standardized mean difference (d) and the odds-ratio (OR). For continuous predictor variables, we used means, standard deviations, and sample sizes (by cell) to calculate the standardized mean difference (*d*). For dichotomous predictor variables, we used sample sizes (by cell) and chi-square values to calculate the odds-ratio. We determined effect size magnitudes following recommendations by Cohen [43] as follows: For the standardized mean difference (d) a value of 0.20 is a small effect, 0.50 is a medium effect, and 0.80 is a large effect. For the odds ratio (OR) a value of 1.50 is a small effect, 2.50 is a medium effect, and 4.30 is a large effect.

## Results

### HIV/Any STI

Table 1 shows results for the primary outcome six-months HIV and/or any STI incidence density by condition and site (n = 1,005). In five of the sites, there was a lower incidence density in the intervention group as compared to the control group in Veracruz, Iguala, Ixtaltepec, Revolución, and Nezahualcoyotl. We categorized these sites as “program effective.” In eight sites that were categorized as “program ineffective” (Naranjos, Tuctla, San Luis Potosí, San Luis de la Paz, Tepeji del Río, Guadalajara, Huajuapan, and Tlapa), the incidence density was significantly lower in the control group compared to the intervention group.

**Table 1:** HIV/Any STI incidence density by condition and site among FSW six months post-randomization to *Mujer Segura* intervention or control group (n = 1055) (2011–2014).

	Control Group		Intervention Group	
Site	Incidence density per 100 person years	95% CI	Incidence density per 100 person years	95% CI
***Program Effective Sites***				
Veracruz	16.12	(0.32, 31.93)	4.54	(0.00, 13.45)
Iguala	38.09	(9.87, 66.31)	4.65	(0.00, 13.78)
Ixtaltepec	21.90	(0.44, 43.37)	6.10	(0.00, 18.05)
Revolución	14.89	(0.00, 35.51)	10.20	(0.00, 24.34)
Nezahualcoyotl	36.09	(9.35, 62.82)	20.54	(0.41, 40.68)
***Program Ineffective Sites***				
Naranjos	11.27	(0.00, 26.90)	11.50	(0.00, 27.44)
Tuxtla	4.80	(0.00, 14.20)	13.06	(0.00, 27.85)
San Luis Potosí	9.20	(0.00, 21.95)	14.25	(0.00, 30.38)
San Luis de la Paz	4.61	(0.00, 13.63)	15.32	(0.00, 32.65)
Tepeji del Río	15.04	(0.00, 32.07)	21.15	(0.42, 41.87)
Guadalajara	10.83	(0.00, 25.84)	27.27	(3.366, 51.17)
Huajuapan	17.06	(0.00, 36.36)	33.58	(6.710, 60.45)
Tlapa	41.79	(10.83, 72.74)	76.70	(35.01, 118.40)
***All Sites***	18.07	(12.79, 23.35)	19.05	(13.66, 24.43)

To further show that program effective sites showed predicted changes in biological and behavioral outcomes, for each site group separately, we also evaluated the effect on the intervention on the 6-month HIV/STI incidence rate and the number of unprotected sex acts with clients during the previous 30 days (reported at 6 months). To evaluate the effect of intervention on the HIV/any STI incidence rate, we used mixed effects Poisson regression with empirical variance estimation and site level random intercepts. Results showed a significant difference in the incident rate ratio between the control groups vs. the intervention groups among program effective sites (IRR = 2.81, 95% CI: 1.30 to 6.05, *t* = 3.73, *p* = 0.02). For the program effective sites combined, the HIV/any STI incidence in the control group was 2.8 times that of the intervention group. There was also a significant difference in the incident rate ratio between the control groups vs. the intervention groups among program ineffective sites. (IRR = 0.53, 95% CI: 0.44 to 0.65, *t* = -7.88 *p* < .001). For the program ineffective sites combined, the HIV/any STI incidence in the control group was 0.5 times that of the intervention group.

To assess the effect of intervention on the number of unprotected sex acts, we used negative binomial regression with empirical variance estimation via generalized estimating equations (GEE). Site was used as a cluster variable with an exchangeable correlation structure (i.e., an identical correlation was assumed between any two participants who belonged to the same site). The number of unprotected sex acts at visit 1 was used as a covariate. For the program effective sites combined, at 6-months of follow-up, the 30 days rate of unprotected sex acts in the intervention group was 0.47 times the corresponding rate in the control group (OR = 0.47, 95% CI: 0.24 to 0.91, Wald χ^2^ = 5.02, *p* = 0.03). For the program ineffective sites combined, there was no significant difference between the intervention and control groups in unprotected sex acts (OR = 070, 95% CI: 0.38 to 1.28, Wald χ^2^ = 1.32, *p* = 0.25).

### Program effective vs. program ineffective sites: Client-related characteristics

Table 2 shows the comparison between FSW from program effective and program ineffective sites on client characteristics (n = 1088). The table summarizes descriptive statistics for each individual variable we tested, the statistical significance, and the effect sizes (Cohen’s d or Odds Ratios). There were statistically significant differences between program effective and ineffective sites on HIV/AIDS-related knowledge (p = 0.03), accompanied by a medium to large effect size (d = 0.55). Women in program effective sites reported greater HIV/AIDS-related knowledge than women in program ineffective sites. We did not find statistically significant differences on any other client-related factors.

**Table 2:** Comparing *Mujer Segura* program effective and ineffective on client-related factors (total n of 1088 FSW) (2011–2014).

	Program Effective Sites (n = 396)		Program Ineffective Sites (n = 692)				
Variable	M	SD	M	SD	*p*	Effect Size	Effect Size Magnitude
Age	33.32	9.84	32.35	9.61	0.55	d = 0.10	< Small
Married (n/%)	97	24.5%	200	28.9%	0.10	OR = 0.80	< Small
Age when first started trading sex	26.18	8.17	26.51	8.19	0.85	d = -0.04	Zero
Hazardous drinking (n/%)					0.72	OR = 1.07	Zero
Yes	307	77.5%	528	76.3%			
No	45	11.4%	121	17.5%			
Used heroin, cocaine, and/or meth, past month (n/%)					0.37	OR = 1.40	< Small
Yes	47	11.9%	61	8.8%			
No	349	88.1%	631	91.2%			
Frequency of using alcohol before/during sex with client, past month (1 = never to 4 = always)	2.05	.92	1.89	0.87	.68	d = 0.18	< Small
Frequency of using drugs before/during sex with client, past month (1 = never to 4 = always)	1.11	0.38	1.06	0.24	.09	d = 0.17	< Small
Rating of overall (poor) financial situation (1 = extremely good to 5 = extremely bad)	3.16	0.67	3.18	0.69	.85	d = -0.03	Zero
Rating of overall (poor) working conditions (1 = extremely good to 5 = extremely bad)	3.00	0.64	2.93	0.69	.11	d = 0.10	< Small
Total number of clients, past month	41.18	52.88	45.82	60.87	0.72	d = -0.08	Zero
Total number of unprotected vaginal and anal sex acts with clients, past month	28.10	39.00	24.71	37.86	0.80	d = 0.09	Zero
HIV/AIDS-related knowledge (items correct out of 18)	14.10	2.33	12.74	2.52	.03*	0.55	Medium to large
Self-efficacy for condom use (1 = strongly disagree to 4 = strongly agree)	2.94	0.57	2.81	0.45	.09	0.26	Small to medium
Positive outcome expectancies for condoms (1 = strongly disagree to 4 = strongly agree)	2.69	0.51	2.56	0.46	.17	0.27	Small to medium
Attitudes towards condom use (1 = very bad to 5 = very good)	4.25	0.82	4.24	0.74	0.97	0.01	Zero

Notes

*p < .05

### Program effective vs. program ineffective sites: Provider-related characteristics

Table 3 shows the comparison between program effective and program ineffective sites on provider characteristics (n = 130). There were no statistically significant differences on any provider characteristics.

**Table 3:** Comparing *Mujer Segura* program effective and ineffective sites on provider-related factors (total n of 130 clinic staff members) (2011–2014).

	Program Effective Sites (n = 47)		Program Ineffective Sites (n = 83)				
Variable	M	SD	M	SD	*p*	Effect Size	Effect Size Magnitude
***Evidence-Based Practice Attitudes Scale***							
Appeal of evidence-based practice	3.03	0.89	2.80	0.81	0.07	d = 0.27	Small to medium
Requirement to adopt evidence-based practice	2.89	0.69	2.73	0.88	0.24	d = 0.19	< Small
Openness to evidence-based practice	2.93	0.60	2.68	0.93	0.14	d = 0.30	Small to medium
Divergence from evidence-based practice (Reversed)	3.39	0.63	3.23	0.65	0.66	d = 0.25	Small to medium
***Emotional Competence Inventory***							
Self-awareness	2.71	0.43	2.58	0.64	0.14	d = 0.23	Small to medium
Self-management	2.53	0.44	2.44	0.59	0.37	d = 0.17	< Small
Social awareness	2.86	0.45	2.72	0.65	0.23	d = 0.24	Small to medium
Social skill	2.65	0.46	2.54	0.69	0.25	d = 0.18	< Small
***Fidelity (n = 515) (193 effective*, *322*, *ineffective)***					0.10	OR = 1.87	Small to medium
Yes (participant said that counselor completed all intervention tasks)	154	77.3%	208	64.6%			
No	39	20.2%	114	35.4%			

Notes: Response scales for for evidence-based practice attitudes and emotional competence was 0 = not at all to 4 = to a very great extent

### Program effective vs. program ineffective sites: Organization-related characteristics

Table 4 shows comparisons between program effective and program ineffective sites on organization characteristics (n = 130). Statistically significant effects were found for organizational social context, specifically in proficiency (p = 0.05) and engagement (p = 0.04), both accompanied by a small to medium effect size. In program effective sites the staff were more proficient (d = 0.38), and were more engaged with clients (d = 0.36) than the staff in program ineffective sites.

**Table 4:** Comparing *Mujer Segura* program effective and ineffective sites on organization-related (total n of 130 clinic staff members) and structure-related factors (total n of 13 sites) (2011–2014).

	Program Effective Sites (n = 47)		Program Ineffective Sites (n = 83)				
Variable	M	SD	M	SD	*p*	Effect Size	Effect Size Magnitude
***Organization -Related Characteristics***							
***Evidence-based practice staffing***	2.69	0.25	2.55	0.46	0.53	d = 0.35	Small to medium
***Evidence-based practice recognition***	1.65	0.93	1.47	0.87	0.36	d = 0.56	Medium to large
***Multifactor Leadership (Transformational Leadership)***							
Individualized consideration	2.82	0.70	2.48	0.86	0.09	d = 0.42	Small to medium
Intellectual stimulation	2.56	0.77	2.34	0.84	0.41	d = 0.27	Small to medium
Inspirational motivation	2.99	0.59	2.78	0.86	0.45	d = 0.27	Small to medium
Idealized influence	2.64	0.62	2.36	0.76	0.11	d = 0.39	Small to medium
***Organizational Social Context***							
Rigidity	1.79	0.51	1.77	0.56	0.81	d = 0.04	Zero
Proficiency	3.02	0.42	2.79	0.70	0.05*	d = 0.38	Small to medium
Resistance	2.17	0.51	2.04	0.60	0.32	d = 0.23	Small to medium
Engagement	1.92	0.23	1.77	0.49	0.04*	d = 0.36	Small to medium
Functionality	2.43	0.49	2.49	0.59	0.58	d = -0.11	< Small
Stress	0.98	0.51	0.91	0.38	0.45	d = 0.16	< Small
Morale	3.09	0.47	2.93	0.60	0.14	d = 0.29	Small to medium
***Structure-Related Characteristics***	**Program Effective Sites (n = 5)**		**Program Ineffective Sites (n = 8)**				
Population total	9.40	8.58	7.99	10.40	0.81	d = 0.14	< Small
Health Index	0.94	0.02	0.91	0.06	0.37	d = 0.61	Medium to large
Education Index	0.85	0.04	0.82	0.06	0.39	d = 0.56	Medium to large
Income Index	0.78	0.05	0.75	0.08	0.36	d = 0.43	Small to medium
Marginalization Index	12.50	7.14	16.54	9.30	0.43	d = -0.47	Small to medium

*Notes*: Response scale for all of the organization-related measures was 0 = not at all to 4 = to a very great extent

### Program effective vs. program ineffective sites: Structural-related characteristics

The bottom of Table 4 shows the comparison between program effective and program ineffective sites in regard to system level regional characteristics (n = 13). The variables were meant to characterize the different clinic sites where the intervention was implemented. Due to the fact that implementation occurred in only 13 sites, the sample size and consequently statistical power are substantially limited [34]. There were no statistically significant differences in structure-related characteristics. On the basis of effect sizes, which should be interpreted with caution in the absence of statistical significance, there were medium to large effect sizes for both the health (d = 0.61) and education (d = 0.56) indices. There were small to medium size effect sizes for income (d = 0.43) and marginalization (d = -0.47) indices. Program effective sites appeared to be located in regions with better health and education infrastructure, and in regions with higher income and lower marginalization compared to program ineffective sites. Again, these results should be interpreted with caution in the absence of statistical significance.

## Discussion

The present study examined the effectiveness of an efficacious HIV prevention intervention (*Mujer Segura*) among FSW in 13 clinics throughout Mexico. We found that in five sites, HIV/STI incidence density was lower in the intervention group as compared to the control group, and we categorized these sites as “program effective” in showing intervention program effectiveness. In eight sites that were categorized as “program ineffective” the incidence density was lower in the control group compared to the intervention group. We also examined different client-, provider-, organization-, and structure-related characteristics as potential correlates of *Mujer Segura* effectiveness. For client-related factors, we found that FSW in program effective sites reported greater HIV/AIDS knowledge than FSW in program ineffective sites. We did not find any statistically significant differences between the two site groups on provider-related factors (e.g., emotional competence). For organization-related factors, we found the program effective sites has staff that reported greater proficiency and engagement with clients than staff in program ineffective sites. For structure-related factors we were limited in statistical power (n = 13). However, effect size magnitudes suggest that regions with better health and education were more likely to have a program effective implementation site. Overall, across all of the variables studied here, we failed to reject the null hypothesis that program effective and ineffective sites were statistically significantly different from one another. We only found few client-, provider-, organization-, and structure-related factors that were associated with effectiveness of our evidence-based program.

Although client variables have been considered central to intervention outcomes in efficacy trials, they have been largely ignored in effectiveness trials [5]. Our findings suggest that future studies should seek to identify the types of client variables that have the greatest impact on intervention program effectiveness. While this study examines effectiveness, these findings suggest that implementation strategies might focus on approaches to improve or enhance client attitudes toward HIV prevention interventions as a component of the implementation process.

A substantial body of literature has also documented the importance of organizational social context in relation to successful adoption and implementation of evidence-based programs [16,44]. The present study showed that staff in program effective sites reported positive attributes that characterize their work environment (e.g., proficiency, engagement with clients). Directors and administrators of organizations would be well advised to work toward developing strategies that foster these attributes among staff in the work environment.

Although structural factors associated with implementation outcomes have been proposed [4,5], few studies have provided empirical evidence of their impact on intervention effectiveness. Findings from the present study suggest that community characteristics may influence how both clients and providers respond to HIV prevention interventions. It appears that income and education inequality as well as health inequalities may hinder effectiveness of HIV interventions for FSW. While not specifically tested in this study, these findings beg the question of the degree to which local context is important to the process of adopting and implementing evidence-based programs for HIV prevention [5]. Future studies should attempt to examine the underlying mechanisms that link specific structural factors to effectiveness of evidence-based programs for FSW and other disadvantaged groups in LMIC.

Overall, we examined many different client-, provider-, organization-, and structure-related variables and how they might be associated with *Mujer Segura* program effectiveness with FSW throughout Mexico. However, only a few of the variables tested were significantly associated with effectiveness. Reasons for this may have to do with the fact that an implementation study of this kind has never been conducted with this population (FSW), and in this region (Mexico). The provider- and organization-related measures included in this study were written and developed in a higher income country- the U.S. For example, the emotional competence inventory was developed in the U.S. using a sample of people with advanced education and skills (e.g., managers in industrial corporations, graduate students in engineering) [34]. It may be that other provider- and organization-related factors not measured in this study are more predictive of program effectiveness, and future research should seek to identify and test such measures. With particular relevance to community/clinic staff working with FSW as their clients, factors like stigma, or attitudes towards women and sex workers may be more relevant to the implementation process and to program effectiveness than factors like transformational leadership.

### Limitations

This study was conducted to examine client, provider, clinic, and system level predictors of effectiveness of an evidence-based HIV prevention intervention with FSW across Mexico. The present findings may lack generalizability to other FSW in other parts of the world, or to other types of interventions. The measure of intervention fidelity may be biased by the participants’ general attitudes towards their counselors. However, using the same fidelity measure we published data showing how fidelity is related to behavior change, suggesting good predictive validity for the measure [2]. We did not include other types of factors that may also facilitate or hinder implementation and effectiveness of *Mujer Segura*, including socio-political factors (police policies, community empowerment) and government funding [45,46].

To our knowledge, this is the first study to systematically examine implementation of a program of this kind in Mexico, and more research is needed to develop new measures to directly and systematically assess the role of providers, organizations, and structural contexts in affecting program effectiveness. Until then, our data help to generate hypotheses that could be tested in future implementation and effectiveness studies. Unfortunately, substantial resources would have to be spent to be able to have the statistical power (i.e., many sites) to detect structure-level differences in program effectiveness and implementation differences across the sites.

## Supporting information

S1 File (DOC)

S2 File (DOC)
