# Supplementary material for: Factors associated with program effectiveness in the implementation of a sexual risk reduction intervention for female sex workers across Mexico: Results from a randomized trial
Source: PLoS One. 2018 Sep 11;13(9):e0201954. doi: 10.1371/journal.pone.0201954 (PMC6133277; doi:10.1371/journal.pone.0201954)
Supplement: S2 File — (DOC) [file pone.0201954.s002.doc]

**UCSD Human Research Protections Program**

**New Biomedical Application**

**RESEARCH PLAN**

Version Date 3/30/2004

(Enter text in the **white space areas** below each numbered heading bar. **Expand the size of table cells as needed** – to multiple pages if needed.

See accompanying Instructions for explanation of headings and information to be provided)

1. PROJECT TITLE

**Implementation of an efficacious intervention for high risk women in Mexico**(NIH R01 MH087054-01A1)
*Revised November 6, 2015*

2. PRINCIPAL INVESTIGATOR

Thomas L. Patterson, Ph.D., Professor in Residence, Dept. of Psychiatry

3. FACILITIES

***Coordinating Center***

This project will be coordinated from Dr. Patterson’s office in SCRB Room 252, SOM Campus, La Jolla.

***Intervention Study Sites***

The *Mujer Segura* implementation study will be carried out at 12 clinics in Mexico that are owned and operated by the Mexican Foundation for Family Planning (MEXFAM), a non-profit, community-based organization that has a registered IRB (#IRB00006816) and an FWA (#00014021). MEXFAM’s central administrative office is located at Juárez 208, Tlalpan, México D.F. 14000, México. The clinics are located in the following cities and states:

• Asunción Ixtaltepec, Oaxaca

• Atizapán de Zaragoza,
Mexico State

• Cd. Nezahualcóyotl, Mexico State

• Guadalajara, Jalisco

• Huajuapan de León, Oaxaca

• Iguala, Guerrero

• Mexico City (Del. Cuauhtémoc)

• Morelia, Michoacán

• San Luís de la Paz, Guanajuato

• Tlapa de Comonfort, Guerrero

• Veracruz, Veracruz

• Zacatecas, Zacatecas

More detailed information on each clinic may be found at http://www.mexfam.org.mx.

4. ESTIMATED DURATION OF THE STUDY

Five years (2010–2015)

5. SPECIFIC AIMS

This project builds upon Dr. Patterson’s earlier study, “Safer Sex Intervention for Female Sex Workers in Mexico” (Project #051182). In that study, we developed a brief behavioral intervention to promote condom use and enhance safer-sex negotiation skills among FSWs in Mexican border cities. The intervention (named *Mujer Segura* in Spanish) was recently demonstrated to be efficacious, mediated by improvements in FSW self-efficacy (see “Progress Report”).

As a result, the federal government in Mexico expressed interest in scaling up the intervention across Mexico and encouraged us to conduct a formal evaluation of the implementation process in order to learn how most effectively to implement this type of intervention throughout Mexico. Therefore, we propose to test a train-the-trainer model for transporting *Mujer Segura* into existing Community Based Organizations (CBOs) in Mexico. This model seeks to develop local and culturally relevant expertise and infrastructure to further disseminate and maintain *Mujer Segura* within each CBO. We will examine whether this model can develop a network of HIV/STI prevention services for FSWs with self-sustaining levels of model fidelity and provider competency. Levels of fidelity to the intervention protocol as well as the efficacy of the intervention itself will be measured at each stage to see how well fidelity and efficacy are preserved through the process.

Our specific aims and corresponding hypotheses are as follows:

Aim 1: To determine if the train-the-trainer implementation model can achieve and sustain high levels of intervention model competency and fidelity.

Hypothesis 1. Competency and fidelity as measured by direct observation methods and FSWs’ reports will achieve at least 90%.

Aim 2: To characterize the relationship between individual provider characteristics and organizational factors, and their impact on the implementation of our intervention, using a mixed-methods (quantitative/qualitative) approach.

Hypotheses 2a –2f: Specific quantitative hypotheses to be examined include:

2a) More positive organizational culture and climate will be associated with more positive attitudes toward evidence-based interventions (EBI).

2b) Personal dispositional innovativeness will mediate the relationship between staff demographics and attitudes toward EBI. Specifically, staff demographics (i.e., higher education level, shorter job tenure) will be associated with higher personal dispositional innovativeness, and personal dispositional innovativeness will be positively associated with attitudes toward EBI.

2c) Social networks indicating more connectivity and positive information regarding *Mujer Segura* will be positively associated with attitudes toward EBI.

2d) Attitudes toward EBI will meditate the relationship between personal dispositional innovativeness and implementation fidelity. Specifically, personal dispositional innovativeness will be positively associated with attitudes toward EBI, and attitudes toward EBI will be positively associated with implementation fidelity.

2e) More positive attitudes toward EBI will be associated with greater implementation fidelity.

2f) Greater fidelity will be associated with more positive FSW outcomes.

Aim 3: To determine whether the implementation of *Mujer Segura* by each CBO is associated with decreased sexual risk behaviors among Mexican FSWs over a six-month period (e.g., increased condom use with clients, reductions in STI incidence);

Hypothesis 3. As in the completed efficacy study, FSWs randomly assigned to the theory-based *Mujer Segura* intervention condition will show greater reductions in high risk sexual behavior with clients (i.e., fewer unprotected vaginal and anal sex acts, lower global STI incidence) at six-month follow-up as compared to FSWs who receive standard counseling.

Aim 4: To determine whether improvements in FSWs’ sexual risk behaviors are associated with variations in intervention fidelity and counselor competency.

Hypothesis 4. Among FSWs in the *Mujer Segura* condition, improvements in sexual behavior with clients at six-month follow-up (i.e., frequency of unprotected vaginal and anal sex, STI incidence) will be associated with higher levels of counselor competency and fidelity to the implementation model.

6. BACKGROUND AND SIGNIFICANCE

Our research with FSWs suggests that HIV prevalence is rising rapidly among FSWs on the Mexico-U.S. border. In a sample of 415 FSWs in Tijuana in 1991, HIV prevalence was 0.5%, but in our more recent study of 924 FSWs in both Tijuana (bordering San Diego, CA) and Ciudad Juarez (bordering El Paso, TX), HIV prevalence was 6%, and HIV incidence in the control group of our subsequent intervention study was 2 per 100 person-years (py). In the same study, prevalence of gonorrhea, Chlamydia and active syphilis was 6.4%,13% and 14.2%, respectively (see Patterson et al. in Bibliography).

As described above in Specific Aims, our *Mujer Segura* brief behavioral intervention proved efficacious in lowering sexual risk behavior and the incidence of HIV and STIs relative to FSWs who received a standard, time-equivalent didactic counseling session. As a result, the federal government in Mexico has expressed interest in scaling up the intervention across Mexico and encouraged us to conduct a formal evaluation of the implementation process in order to learn how more effectively to implement this type of intervention throughout Mexico.

Several meta-analytic reviews have identified efficacious interventions for reducing high-risk sexual behaviors in a variety of populations, including Men who have Sex with Men (MSM), ethnic minorities, and HIV+ individuals. However, there has been a lack of empirical study of the implementation of evidence-based HIV interventions (EBIs) to community-based organizations (CBOs). To encourage the adoption of EBIs by CBOs, the Centers for Disease Control (CDC) in 2002 implemented the Diffusion of Effective Interventions (DEBI) Project, which was designed to implement evidence-based interventions in community-based programs. The experiences of DEBI participants form the basis for our understanding of key issues relevant to translating research into practice.

Both client and provider characteristics are important in terms of achieving a good fit between the EBI and the implementation agency. *Mujer Segura* is a brief (35-minute) intervention that meets the needs of FSWs in Mexico. Because this is a resource-poor population, FSWs will not accept a program that involves a major time commitment that would interfere with income earning. *Mujer Segura* also offers clients free HIV and STI testing, which is valued by the participants. FSWs are a diverse group in terms of age and years of work experience; thus, women are at different stages in the behavioral change process, which is taken into account in the protocol (stages of change theory is incorporated into the motivational interviewing component). Also, the one-on-one counseling element is appealing to FSWs because many are not willing to participate in group sessions. *Mujer Segura* is also a good fit with the CBOs that are willing to adopt this intervention. “Values–innovation fit” is defined as the fit between the values an organization or service provider implementing an innovation and the characteristics of the innovation itself and is important in the implementation of innovations such as *Mujer Segura*. Data gathered from the directors of the MEXFAM CBOs indicate that *Mujer Segura* is compatible with MexFam’s philosophy and mission. The intervention was also perceived as feasible, relevant to local culture, sustainable, and able to fill a gap in existing HIV prevention services for FSWs in resource-constrained settings (see below, Preliminary Studies).

7. PROGRESS REPORT/PRELIMINARY STUDIES

***Mujer Segura Intervention Trial Results***

Results of the first phase of *Mujer Segura* (NIH R01 MH065849, Project #051182) were described in the following article (see Appendix 1):

Patterson, T.L., Mausbach, B.T., Lozada, R., Staines, H., Semple, S.J., Fraga, M., Orozovich, P., Abramovitz, D., de la Torre, A., Amaro, H., Martinez, G., Magis-Rodríguez, C., and Strathdee, S.A. Efficacy of a brief behavioral intervention to promote condom use among female sex workers in two Mexican-u.s. border cities. *American Journal of Public Health.* 98 (11): 2051-7, 2008.

After participating in our 35-minute theory-based intervention, FSWs randomized into *Mujer Segura* had a 40% decline in cumulative STI incidence (p=0.03). Incidence density for the intervention vs. control was 13.8 vs. 24.92 per 100 person-years (py) for STIs combined (p=0.03), and 0 vs. 2.01 per 100 py for HIV (p<0.001), with concomitant increases in total numbers and percentages of protected sex acts and decreases in total numbers of unprotected sex acts with clients (p<0.05) at six-month follow-up. These results establish a baseline for efficacy against which successive implementations of *Mujer Segura* at other sites within Mexico can be measured.

Our human-subjects experience with *Mujer Segura* was excellent. A total of 1,853 participants were recruited, and none complained about any aspect of her treatment. Participant confidentiality was effectively maintained throughout the study. This was due to thorough training of staff at all implementation sites on the importance of informed consent and participant confidentiality. The same training measures will be implemented in the present study at all implementation sites.

***Selection of CBOs at which to Study the Implementation of the Intervention***

With the assistance of Dr. Carlos Magis, a consultant on this project and Director of CENSIDA (the Mexican federal agency for the control and prevention of HIV and AIDS), we identified a community-based, non-profit organization that is both willing and able to provide the facilities and staff that we require in order to implement our study. MexFam has a total of 20 CBOs in 19 Mexican states. We excluded eight clinics from further consideration based on the following criteria: based in a rural community, clinic population does not include FSWs, clinic program is focused on children under the age of 18, or clinic is based in a city that participated in the original *Mujer Segura* project. Based on these exclusion criteria, 12 MexFam CBOs in 12 cities in Mexico were evaluated and determined to possess the qualities considered optimal for participation (see list in Section 3, above).

Each CBO selected for participation has been deemed to have the following characteristics: 1) a core of eight high-potential, stable staff members who will be trained as *Mujer Segura* counselors; 2) an organizational culture that supports innovation and evidence-based perspectives; 3) strong local reputation and street credibility; 4) capacity to deliver professional peer-to-peer training; 5) strong cultural competency and knowledge about FSW populations.

8. RESEARCH DESIGN AND METHODS

Data collection in this study will occur on two levels: (1) a mixed, quantitative and qualitative study of the organizations (CBOs) that will implement the *Mujer Segura* intervention, and (2) behavioral and health outcomes for the female sex workers (FSWs) who will participate in the intervention at each CBO.

***The Implementation Process: Organizational Level***

The implementation plan embraces four fundamental principles:

1) Developing local program expertise is important for sustaining model competency and fidelity. Involvement of model developers and practice experts should be front-loaded, then roles shift to local experts as they are developed.

2) Direct coaching and modeling in the actual practice setting is critical for optimal provider skill acquisition.

3) Maintaining and monitoring fidelity across time is critical. Adaptations to the local context or evolutionary changes in the model should be carefully planned, transparent and systematically implemented rather than made idiosyncratically or haphazardly.

4) Policy leadership and funding structures are important for institutionalizing the new practice model.

Phase One: Evaluation of Organizational Characteristics and Training of Staff and Delivery of Comparison Condition Intervention. Two trained ethnographers from the Ciudad Juárez training center will administer computer-based questionnaires to **ten staff (the director, intervention counselors, and supervisors).** The ethnographers will then conduct individual, semi-structured qualitative interviews with each participating staff member, and finally they will carry out a focus group session with all the participants at once in order to assess relevant provider and organizational factors. The questionnaires, the individual interviews, and the focus group session will all last about an hour each, and they will be tape-recorded. Provider factors to be assessed include: attitudes toward adoption of innovation (e.g., intuitive appeal of intervention program); personal disposition (e.g., adaptability, change catalyst); knowledge and perceived utility of intervention; and socio-demographic characteristics. Organizational factors include: leadership qualities; organization culture and climate (e.g., work attitudes, autonomy); organizational support (e.g., support for creativity); and social influence (e.g., teamwork). The same battery of assessments was used by Dr. Aarons in a study of a children’s service agency (see Aarons, item 5 in Bibliography). A copy of that assessment battery is provided in Appendix 2. The measures have all been translated into Spanish, and the name of the organization has been changed to fit the new study.

To avoid contamination between the experimental and comparison conditions, CBO staff will first evaluate and deliver the “usual care” counseling session to the control group before learning how to deliver the experimental counseling intervention. A total of 80 FSWs will be recruited at each site, of whom 40 will be randomly assigned to the *Mujer Segura* counseling condition and 40 will be assigned to the usual care risk-reduction counseling condition, which was developed by Mexico’s federal HIV-AIDS agency (CENSIDA) and focuses upon basic educational information, personal risk assessment, and strategies for reducing personal risk. Participants who are randomized at intake to the experimental condition will be asked to return at a later date to receive their assessment and intervention.

Phase Two: Training the CBO’s designated “internal trainer.” Once the comparison condition FSWs have been assessed and counseled, a participating CBO staff member will be designated as the CBO’s “internal trainer” and receive intensive training in both the *Mujer Segura* protocol and in effective methods for teaching the protocol to other staff. Drs. Patterson, Staines, and Aguirre and two practice experts from the Ciudad Juarez training center will conduct an intensive, eight-day training.

Phase Three: The “internal trainer” trains CBO staff who in turn deliver the *Mujer Segura* intervention. The practice experts will remain at the CBO for two weeks to observe and coach the internal trainer as she trains the counselors who will deliver *Mujer Segura* to the 40 randomized FSWs, and the counselors will begin to deliver the intervention. The experts will give feedback on the delivery and help the CBO counselors resolve challenging or idiosyncratic case issues or presentations. After the practice experts leave, they will maintain weekly telephone and email contact with the CBO counselors.

While the CBO is implementing *Mujer Segura* (over approximately a six-month period), the ethnographers will return periodically to monitor intervention fidelity. Finally, at the end of implementation, the ethnographers will return to repeat the staff qualitative interviews and focus groups that were performed at the outset, in order to enable the researchers to see if any changes have occurred in staff attitudes or organizational characteristics over the course of implementing the intervention.

Phase Four: Maintenance. In the fourth and final phase of the implementation model, the internal trainer will shift roles to maintenance. Maintenance activities include training and coaching new hires, a lower level of in vivo coaching and fidelity monitoring of existing trainees (quarterly rather than weekly or bi-weekly), and planning any major adaptations or evolutionary modifications of the model.

***The Intervention: Level of the FSW***

A secondary aim of this study is to determine how FSW behavioral outcomes change concordant with the fidelity of the implementation of *Mujer Segura* at each CBO and with other organizational characteristics. FSW data will be gathered at baseline (prior to counseling) and again at six-month follow-up using measures employed in our previous work with Mexican FSWs (Project #051182), which was reviewed and approved by the IRB.

Baseline and Follow-up Interview. The baseline interview (~30 min) is theory-driven and has been administered to over 1,800 FSWs in Mexico-U.S. border cities. Per the recommendations of our Mexican collaborators, all measures will be administered by computer-assisted personal interviewing (CAPI). Reading ability is not a requirement of study participation. Using CAPI technology, the interviewer will read all questions and response categories to the participant and record the participant’s responses on a computerized form. In order to measure intervention effects, the interview will be repeated with each participant at a six-month follow-up appointment. The interview covers the following domains:

• Background Characteristics. These include socio-demographics, family background variables (e.g., number of children), and financial need (e.g., number of financial dependents).

• Contextual factors. Interview questions include working conditions: work setting (e.g., street), type of sex worker (e.g., brothel-based), nature of relationship with pimp or manager (if applicable) (e.g., control over client selection), client characteristics, demands for unprotected sex, amount received for protected vs. unprotected sex, and availability of condoms and sterile syringes.

• Substance Use. History, practices and environmental influences, including age at first use of alcohol and specific drugs, amount of alcohol consumed using the AUDIT; types of substances used alone and in combination, routes of administration, and injection practices.

• Mechanisms of Change. Consistent with our theoretical framework, we include measures of attitudes, intentions, and peer norms about safer injection and sex.

• HIV and STI Knowledge will be measured with an 18-item scale that assesses awareness of the importance of condom use with respect to HIV/STI prevention (alpha = 0.75 to 0.89).

• Self-Efficacy towards Condom Use is a 5-item measure that asks participants the extent to which they are able to use a condom properly with clients, (alpha = 0.85).

• Peer norms about safer sex will be measured by two items developed by Fisher et al. (e.g., “Most people in my line of work think that I should always use condoms for vaginal intercourse” (alpha=0.96).

• Attitudes toward HIV Prevention: Four items will be used to measure attitudes toward HIV prevention (e.g., “My not having (vaginal/anal) intercourse with my clients during the next month would be: very good (5), somewhat good (4), neither good nor bad (3), somewhat bad (2), and very bad (1)” (alpha = 0.79).

• Outcome Expectancies will be measured by 6 items (alpha= 0.89. Sample items are “I believe that using condoms will protect me from getting HIV” and “I don’t think sharing my works will put me at risk for HIV”.

• Sexual Risk Behaviors include number and frequency of unprotected vaginal, oral, and anal sex with clients and with spouse or steady partner(s); number of clients (regular and non-regular); number and type of other sex partners (non-clients); number of partners who inject drugs.

Testing for HIV and STIs. A blood test will be performed at baseline and six-month follow-up to ascertain HIV serostatus. The blood draw (approximately 10 cc) and testing will take place at each participating CBO. FSWs will also receive a gynecological exam that will yield a vaginal smear and gonorrhea culture. Specimen testing will be conducted on-site at each MexFam CBO. The “Determine”® rapid HIV antibody test will be administered to determine the presence of HIV antibodies (Abbott Pharmaceuticals, Boston, MA). All reactive samples will then be tested using HIV-1 antibody by EIA and Western Blot. Syphilis serology used the rapid plasma reagin (RPR) test (Macro-Vue, Becton Dickenson, Cockeysville, MD, USA). All RPR-positive samples will be subjected to confirmatory testing using the *Treponema pallidum* hemagglutinin assay (TPHA) (Fujirebio, Wilmington, DE, USA). *Neisseria gonorrhea* and *Chlamydia trachomatis* samples will be collected by trained nurses using the Aptima® Combo 2 collection device (Genprobe, San Diego, CA), which allows for a direct target-amplified nucleic acid probe test. HIV/STI test results will be provided to participants by nurses within one week of testing. Those testing HIV-positive will be referred to the municipal health clinic in their city for free medical care. Participants who test positive for another STI will be treated on-site at the MexFam CBO.

***Data Analysis and Interpretation***

Aim 1: Equivalence testing will be used to evaluate Aim 1 using Schuirmann’s (1987) test of equivalence. A nominal alpha of .05 will be used in the proposed analysis. Given the nested data structure (i.e., service providers nested in CBOs), hierarchical linear modeling will be used to adjust standard errors for possible dependency among observations. The outcome of interest in Aim 1 focuses on *Mujer Segura* fidelity. We expect that service providers will attain approximately 90% treatment fidelity when considering that 100% is perfect fidelity.

Power calculations for equivalence tests proposed for Aim 1 were conducted using SAS® Proc Power. Using the bounds and nominal alpha specified above, along with a hypothesized difference between population group means of zero and a standard deviation of 15 in each group, the proposed sample size of 120 (10 staff x 12 CBOs) would yield power of >.89 to reject the null hypothesis of mean differences in favor of the alternative of group equivalence. This power calculation is based on the assumption that observations are independent. If the observations are dependent, this will be an overestimate of statistical power. One method of calculating power in this case is to estimate the degree to which the variance will be increased due to non-independence. Specifically, the variance will be increased by a factor of 1+(m-1)*ICC, also known as the design effect, where m is the average cluster size and ICC is the intraclass correlation coefficient. While we don’t have prior ICCs for *Mujer Segura* fidelity ratings, in our work examining service provider attitudes toward EBI implementation in CBOs, ICCs were generally low. However, in a larger and more representative study of CBOs across the U.S., the ICC was .03, and we expect the fidelity ICC to be of similar magnitude. Using the average ICC of .03 from this recent study, and an average cluster size of 8, the variance is estimated to be increased by a factor of 1.21. Given a total sample size of 96, the functional sample size would be 79.34 (i.e., total sample size/design effect), which would yield power of .80.

Aim 2: Analyses will be informed by the conceptual model proposed by Aarons and adapted for this study in which implementation of EBI results in effects of organizational and provider characteristics on provider attitudes, fidelity and outcomes. Conversely, introduction of an EBI can impact organizational and—to a lesser extent—provider characteristics. In particular, we will examine changes in organizational processes (i.e., culture, climate) and group process characteristics (e.g., organizational support) as a function of *Mujer Segura* implementation. We will also examine the relationship between individual organizational factors and provider characteristics, and their impact on the implementation of *Mujer Segura*. We will then examine whether more knowledge and higher levels of perceived utility are associated with better fidelity and outcomes. In all CBOs, we will examine the degree to which Leadership and Organizational Support are associated with more positive Attitudes Toward Evidence-Based Practice, fidelity, and outcomes. We will also examine the degree to which Personal Dispositional Innovativeness and other provider characteristics such as age, education level, experience, and job tenure are associated with Attitudes Toward Evidence-Based Interventions, fidelity, and outcomes. Quantitative hypotheses to be examined include:

2a) More positive organizational culture and climate will be associated with more positive attitudes toward EBI.

2b) Personal dispositional innovativeness will mediate the relationship between staff demographics and attitudes toward EBI. Specifically, staff demographics (i.e., higher education level, shorter job tenure) will be associated with higher personal dispositional innovativeness, and personal dispositional innovativeness will be positively associated with attitudes toward EBI.

2c) Social networks indicating more connectivity and positive information regarding *Mujer Segura* will be positively associated with attitudes toward EBI.

2d) Attitudes toward EBI will meditate the relationship between personal dispositional innovativeness and implementation fidelity. Specifically, personal dispositional innovativeness will be positively associated with attitudes toward EBI, and attitudes toward EBI will be positively associated with implementation fidelity.

2e) More positive attitudes toward EBI will be associated with greater implementation fidelity.

2f) Greater fidelity will be associated with more positive FSW outcomes.

In order to examine components of relationships described in 2a-2f, analyses will use HLM in order to adjust standard errors for possible dependency among observations. Significance tests will use an alpha of .05 (two-tailed). Analyses will consist of correlations (2c, 2e, 2f), regression (2a), and tests of mediation (2b, 2d). Mediation or indirect effects will be tested using confidence intervals. Given that bootstrapping confidence intervals will likely not be an option in this study due to missing data (although missing data will be handled through multiple imputation methods [described above] for bootstrapping from a series of imputed data sets are not available), intervals will be constructed based on the distribution of the product of two random variables. This method of significance testing is superior to non-resampling alternatives in terms of having greater Type I error rate accuracy and higher power. The intervals will be estimated using PRODCLIN.

Power was calculated under the assumptions of independent and dependent observations for the proposed correlation, regression, and mediation tests. Under independence the sample size used was the total of 96 and under dependence (ICC = .03) a functional sample size of 79.34 was used (see Aim1). Power for the correlation hypotheses was evaluated using SAS® Proc Power. The power to detect a medium effect size (r = .30) for correlation hypotheses was calculated to be .85 under independence and .78 under dependence. Power for the regression hypotheses was evaluated using the Monte Carlo facility of Mplus with 20,000 replications. All parameters of the regression model were set to be medium effect sizes based on bivariate association conventions, that is, the correlation between each predictor and the outcome and the correlation among the predictors was set to be *r* = .30. The power for each regression coefficient was calculated to be .79 under independence and .71 under dependence. Finally, for tests of mediation, simulations by Fritz and Mackinnon suggest that the sample size needed for power of .80 using the proposed distribution of the product of two random variables approach (i.e., PRODCLIN) is 74, when the effect sizes for both paths are medium (i.e., $\alpha$=$\beta$=.39). The total N of 96 used under the assumption of independence and 79.34 under dependence will be sufficient to achieve power of .80.

Aims 3 & 4: Due to potential non-independence of FSWs within CBOs, mixed linear models will be used to determine if participation in the *Mujer Segura* intervention was effective in improving sexual behavior outcomes (e.g., condom use, ratio of unprotected to protected sex) compared to the treatment-as-usual condition. Within this model, intervention condition, time, and the intervention-by-time interaction will be entered as our primary independent variables. Because participants will be nested within CBOs, this factor will be entered as a random effect. A first-order autoregressive process (a stationary AR[1] process) will be used to characterize the autocorrelation structure.

Our second set of analyses will examine efficacy of the intervention for reducing incident HIV and other STIs. We will compare intervention groups in terms of cumulative incidence and incidence density. In both cases, the numerator will be the number of women who acquire the STI in question during follow-up; for cumulative incidence, the denominator will be the total number of at-risk women, whereas for incidence density the denominator will be the total number of person years for at-risk women. For each HIV/STI outcome, Poisson regression will be used to determine if group differences are statistically significant. As an indication of effect size, we will calculate the Number Needed to Treat (NNT) for each STI outcome, where NNT represents the number of participants in the intervention who would need to be treated before one fewer person contracted the STI than would be the case had all participants received the comparison condition. Aim 4 will be addressed by using FSW behavioral outcomes described above (aim 3).

We evaluated statistical power to detect main effects and interaction effects in Hypotheses 3 and 4. Our power analyses reveal that we have an excellent chance of detecting significant main effects and interactions in our data. Hypothesis 3 uses a repeated measures design with two conditions. Assuming an attrition rate of 20%, which we achieved in *Mujer Segura*, we will have 384 subjects per group for a total of 768 subjects at the end of five years. We used findings from the parent project intervention with FSWs described in Preliminary Studies to estimate overall reductions in high-risk sex and incident STIs. We observed a 36% reduction in unprotected sex in the intervention (i.e., 18% vs. 36%), and a 40% decrease in overall STI incidence in the intervention group relative to the control (i.e., 20% vs. 38%). To estimate power in our sample, assuming a 36% reduction in unprotected sex at six-month follow-up, we calculated the following: N=384 per group, alpha = .05, and a 36% reduction in mean number of unprotected sex acts. If subjects in our experimental condition reduced the number of unprotected sex acts while the control condition did not change (i.e., group x time interaction), we would detect significant differences greater than 78% of the time. Power is slightly higher for STIs (i.e., .81). The addition of a covariate for fidelity to test hypothesis 4 does not substantially impact power estimates. Therefore, though our study is not designed to replicate findings from the parent project, we have sufficient power to detect differences between groups.

***Qualitative Analyses.*** Qualitative analyses are conceptualized as providing complementary information that will facilitate interpretation of quantitative analyses as well as provide the basis for refinement of measurement in future studies. Qualitative interviews will be recorded and transcribed, then analyzed by Dr. Palinkas and our two ethnographers from Ciudad Juarez. The empirical material contained in the field notes, interviews and focus group sessions will be independently coded by the project researchers to condense the data into analyzable units. Codes will also be assigned to material to reflect the social and demographic characteristics of study participants. Lists of codes developed by each qualitative researcher will be matched and integrated into a single codebook. With the final coding structure, two of our qualitative researchers will separately review transcripts to determine level of agreement in the codes applied. A level of agreement in the codes applied ranging from 66 to 97 percent depending on level of coding (general, intermediate, specific), indicates good reliability in qualitative research. The computer program QSR NVivo will then be used to generate a series of categories arranged in a treelike structure connecting text segments grouped into separate categories of codes or “nodes.” These nodes and trees will be used to further the process of axial or pattern coding to examine the association between different a priori and emergent categories.

9. HUMAN SUBJECTS

There will be two groups of participants in this study: (1) CBO personnel and (2) FSWs recruited into the *Mujer Segura* intervention study.

***CBO Staff***

The primary participants will be CBO personnel, including staff who will deliver the intervention, their supervisors, and local directors (12 CBOs, approximately 10 personnel per site = 120). Twelve CBOs affiliated with MexFam will be enrolled into this project. We expect that some of the smaller clinics may not be able to provide a quota of 10 staff. Accordingly, to ensure that we recruit the desired minimum the entire study, we request flexibility in the per-clinic figures to enable us to recruit more than 10 if opportunity affords, up to a maximum of 135 staff for the entire study.

The CBO staff who participate in the organizational-level portion of this study will be recruited based upon their institutional role. MexFam’s executive leadership will make it clear to all staff that although the intervention study with FSWs is part of MexFam’s mission and hence is something to which all staff will be expected to contribute if necessary, the organizational-level study (individual interviews, focus groups) is entirely voluntary, and that no adverse consequences will follow upon a decision not to participate. A modest financial incentive, over and above the potential participant’s normal salary (which he or she will also receive for the approximately 6 hours total time spent on activities related to the part of the study) will be offered to encourage participation (see Section 18).

***Intervention Study Participants (FSWs)***

The second set of participants in this research will be the FSWs who are recruited at each CBO to participate in the baseline and follow-up assessments and either the *Mujer Segura* counseling intervention or the “usual care” comparison condition. The recruitment goal for the study will be 960 female sex workers (FSWs) from the twelve anticipated sites (12 CBOs times 80 FSWs per site = 960). As with the staff participants (see above), we are uncertain whether the all of the currently projected sites will be able to meet this quota. We also face the uncertainty (particularly in the early phases of the study) whether, once the study has been started at a particular site, the staff will be able to carry it to completion due to factors beyond our or Mexfam’s control. Accordingly, we request flexibility in the per-clinic and overall figures for recruiting FSWs, up to a maximum of 1,090 for the entire study (in increase of 130, which is 13.5% more, or the equivalent of less than two additional sites).

We anticipate that participants will range in age from 18 to 50 years (mean approx. 33); mean level of educational attainment will be 6 years; 80% will be married or living in a common-law relationship; and 94% will have children.

Inclusion Criteria: To be eligible for this intervention, participants must be:

1) Biologically female,

2) At least 18 years old,

3) Report having exchanged sex for money, goods or drugs within the previous 30 days,

4) Report having unprotected vaginal or anal sex with a male client at least once during the previous month.

Exclusion Criteria:

1) Consistent use of condoms for vaginal and anal sex with all male clients during the previous month,

2) Known to be HIV+ or test HIV+ for the first time on-site at MexFam clinic,

3) Being under 18 years of age,

4) Being male or transgendered,

5) Being incapable of giving informed consent,

Because this study is designed to transport an established, efficacious intervention into a community-based setting, we must adhere to core elements of the established intervention, which was designed for women who are biologically female. In the original intervention design, transgendered persons were excluded because this subpopulation of FSWs would require different recruitment strategies and a targeted intervention to reflect unique sexual risk behaviors and sexual milieu.

10. RECRUITMENT

***CBO Staff***

The Executive Director of MexFam or his representative will ensure that, prior to implementation of the study, all eligible clinic staff are given an overview of the project that carefully distinguishes between the intervention study component (which is congruent with MexFam’s mission to protect and improve the health of vulnerable populations and, as such, has a claim on all employees’ involvement as part of their normal job duties) and the organizational-level component of the study, for which the employees’ informed consent is required prior to their enrolling as participants. The Executive Director will ensure that all employees understand that their participation in the organizational-level part of the study is voluntary, and that their employment status or compensation will not be affected by a refusal to participate. The study’s ethnographers will then visit each CBO and invite eligible staff (those who will deliver the intervention, their supervisors, and the clinic’s director) to participate.

***FSW Intervention Participants***

Participants will be recruited from designated areas in which sex work occurs in the city where the CBO is located. Based on our experience, sex work venues include bars, hotels, street corners, massage parlors and brothels. FSWs will be approached in the field by trained outreach workers who will ascertain the participant’s willingness and eligibility to participate in the study through the use of six screening questions (no identifiers on form) asked in a conversational manner. FSWs who are potentially appropriate for inclusion and willing to participate in the study will be referred to the participating MexFam clinic (transportation will be provided as needed).

11. INFORMED CONSENT (Note: provide information in Section 28 on Surrogate Consent and Decisional Capacity Assessment, if applicable)

***CBO Staff***

The study’s ethno­graphers, having identified staff who are willing to participate in the organizational-level component of the study, will review the informed consent document with them, which contains all pertinent information regarding study procedures, risks and benefits, safeguards for confidentiality, and compensation. Potential participants who still have questions after these explanations will be put in touch with Dr. Staines or Dr. Patterson.

***FSW Intervention Participants***

The informed consent procedure is designed to maximize the potential participant’s comprehension of study procedures and to ensure that participation is voluntary. Before a participant is enrolled, the purpose, the procedures to be followed, and the risks and benefits of participation will be explained by project staff, and signed informed consent from the participant will be obtained. To obtain informed consent from women who are illiterate, a two-step solution was suggested by the Director of the UCSD HRPP (Dr. Michael Caligiuri) and UCSD ethicist, Dr. Lawrence Schneiderman: 1) consent forms are read out loud, and 2) a simple test is given wherein participants are questioned about points made in each paragraph of the consent (e.g., “How much time will this take you?”; “What are the possible benefits for you?”). When errors are encountered, the paragraph is re-read and participants are encouraged to ask questions to clarify. They are retested to ensure they understand all passages. If, after further attempts to clarify any misunderstandings, project staff determine that a potential participant may not fully comprehend the critical aspects of the study, that potential participant will not be enrolled. If a potential participant decides she does not wish to participate, her decision will be honored regardless of how well she comprehends the study information. This process worked well through all five years of our *Mujer Segura* study (Project #051182).

12. THERAPEUTIC ALTERNATIVES (therapeutic studies only)

Since the desired primary outcomes of the intervention study with FSWs are increased use of condoms with clients and decreased incidence (i.e. prevention) of HIV and STIs, FSWs who are considering enrollment in the study will be informed in the consent document of alternative methods for decreasing their sexual risk behavior and HIV and STI risk.

Treatment of incident HIV and STIs is not a focus of the study; however, FSWs who enroll and who test positive for HIV or an STI are eligible under Mexico’s national health insurance program to receive free treatment at the municipal clinic in each participating city. All FSWs testing positive will be referred to the appropriate clinic for treatment.

13. POTENTIAL RISKS

***CBO Staff***

The risks likely to be encountered by clinic staff by participating in the one-on-one qualitative interviews and focus-group meetings are minimal. Participants may experience boredom or minor irritation at the nature of some of the questions asked. There is also a slight risk that a subject’s candid responses to questions about the organization may become known to clinic management and that management, contrary to organizational policy, may attempt to retaliate against subjects offering negative assessments of their organization or their employment situation.

In view of recent increases in drug-related violence in various parts of Mexico, CBO staff who go into the field as recruiters may incur an increased risk of finding themselves unexpectedly in harm’s way.

***FSW Intervention Participants***

The FSWs who enroll in the *Mujer Segura* intervention study face the following risks:

1) Bruising or fainting as a result of the venipuncture. These risks are deemed negligible, due to the training and experience of the clinic personnel (nurses, trained phlebotomists) who will perform the venipuncture.

2) FSWs who obtain a positive HIV test may experience the loss of a sex work permit (in localities where such permits are required), stigmatization, loss of private insurance (public health care is available to all Mexicans at no charge), loss of employment, loss of freedom to travel to some countries, loss of relationships with family, spouse, or friends, and anxiety or depression concerning their health, the health of their children, and their sexual partners.

3) Emotional distress due to the personal nature of many of the questions asked during the baseline and follow-up interviews.

4) Loss of confidentiality (i.e., a participant’s involvement of the study and hence her occupation as an FSW becomes known outside the study), which could result in loss of reputation or stigmatization. The risk of loss of confidentiality is deemed minimal based upon our experience with over 1,800 FSWs in our *Mujer Segura* intervention study (Project #051182), which used the same protocol and experienced no known breaches of confidentiality.

5) There is also some risk that counselors will receive reports of physical abuse and violence perpetrated against FSWs by clients or other sex partners. This is not a risk of the protocol per se, but rather an incidental result of it.

14. RISK MANAGEMENT PROCEDURES

***CBO Staff***

The risks of boredom or irritation during one-on-one interviews or focus group meetings have been minimized by keeping the length of each session to one hour or less. These risks will further be minimized by having the interviewer or focus-group leader assure the participants that they may refuse to answer any questions to which they object, and that they may request a short break at any time.

The risk of retaliation by clinic management for negative evaluations of organizational characteristics will be minimized in two ways: (1) MexFam’s Executive Director will clearly instruct all clinic directors that participant candor is to be encouraged and that no participant shall be penalized in any way for expressing his or her opinions, and (2) the organizational data (both quantitative and qualitative) will gathered and handled exclusively by ethnographers from the Cd. Juarez site, which is independent of MexFam. These data will not be shared with MexFam management unless the data have been de-identified both as to individuals and as to the site where the data were collected.

**Digital audio-recordings gathered from interviews with MexFam staff (counselors, directors, supervisors) will be stored on password-protected computers and identified by ID number only. Recordings will be transcribed as soon as possible and will be destroyed within two months after transcription. Counselor notes and worksheets will contain ID numbers only. These forms will be shredded at the completion of six-month follow-up. The key to participants’ code numbers (name-to-ID relational file) will be encrypted in a computer file, which will be locked in the director’s office at each MexFam CBO and in Dr. Patterson’s office in San Diego. Only the CBO directors, Drs. Patterson and Semple, and the USCD data manager will be able to un-encrypt the computer file. Other security mechanisms at the MexFam CBOs and UCSD include: security workshops and written security policies and procedures.**

**The risks of inadvertent exposure on the part of recruiters to crime-related violence in the field will be addressed by ensuring that Mexfam’s existing security policies are carried out, which require that all new employees receive training in safety awareness and security procedures. Should conditions in the area around any of the Mexfam clinics give rise to increased concerns, the clinic director, in consultation with Mexfam’s executive director, will revise security procedures as necessary and promptly brief his or her employees.**

***FSW Intervention Participants***

1) Risks from venipuncture will be minimized through the use of trained medical personnel (nurses, phlebo­to­mists). Each MexFam CBO that participates in this project is a well-established community clinic that conforms with all applicable state health regulations regarding facilities, supplies, and the qualifications of clinic staff.

2) All FSWs who receive a positive HIV test result will receive counseling from trained clinic personnel and referrals to medical and psychological support services. It should be noted that the FSW population is accustomed to regular HIV and STD tests in a clinical setting. MexFam clinics routinely offer HIV and STI counseling to clients, and staff have ample experience in addressing the immediate psychological and referral needs of clients with regard to these conditions.

3) Drs. Patterson and Staines have more than 25 years of combined experience training staff to conduct confidential and sensitive interviews regarding highly personal information. All staff in MexFam CBOs will be trained to approach questions with sensitivity and in a supportive manner.

4) To guard confidentiality, all questionnaire data will be in stored in computerized form with ID numbers only (no names). Computers will be stored in locked offices at MexFam CBOs and UCSD. **Contact information for enrolled participants (locator form) will be stored in a locked file cabinet in the director’s office at each CBO; the locator form will not have the participant’s identifier number. Locator forms will be shredded at the completion of six-month follow-up, unless the participant has indicated on her consent form that she is willing to be contacted for additional studies; in this case, the locator form will be retained under secure conditions for an additional two years and shredded at the end of that period. Original signed consent forms will be stored in a locked file cabinet in the Project Official’s office at MexFam in Mexico City. A copy of all consent forms will be shipped to San Diego for permanent storage in a locked file cabinet in Dr. Patterson’s office. Digital audio-recordings of intervention sessions with FSWs made for training and quality-assurance purposes will be stored on password-protected computers and will not be labeled with any information that identifies the FSW. The key to participants’ code numbers (name-to-ID relational file) will be encrypted in a computer file, which will be locked in the director’s office at each MexFam CBO and in Dr. Patterson’s office in San Diego. Only the CBO directors, Drs. Patterson and Semple, and the USCD data manager will be able to un-encrypt the computer file. Other security mechanisms at the MexFam CBOs and UCSD include: security workshops and written security policies and procedures.**

**5) Participants in the behavioral intervention study who report being victims of physical, emotional, or sexual abuse** will be referred to an on-site social worker. Dr. Patterson has discussed the potential for violence with the Director of UCSD’s HRPP. The HRPP’s policy is that violence that is related to the conduct of the protocol must be reported to the IRB as an Unanticipated Problem Involving Risk to Participants or Others (UPR). Violence unrelated to the protocol that is incidentally communicated during the conduct of the protocol may be reportable to civil authorities under applicable Mexican local law if the woman so chooses, but it would not be reportable as a UPR. In the event of violence related to our intervention, an incident report will be filed with both the UCSD IRB and the MexFam IRB. If, during the course of the interview or counseling, the participant reveals current abuse, neglect, or planned danger to self or others, the staff member will report this to the MexFam CBO director, who will make the appropriate referral as required by Mexican law. The incident will also be reported to MexFam’s Executive Director (Dr. Vicente Diaz) and Dr. Patterson within 24 hours. Dr. Diaz will inform the MexFam IRB and Dr. Patterson will file a report with the UCSD IRB.

***Data Safety and Monitoring Board***

Dr. Patterson’s affiliation with the UCSD HIV Neurobehavioral Research Center (HNRC) provides access to its Data Safety and Monitoring Board (DSMB), which has been designed to ensure the safety and welfare of participants and the validity and confidentiality of data. The board’s responsibilities include: reviewing protocols, informed consent documents, and plans for data safety and monitoring, evaluating the progress of intervention trials, participant risk versus benefit, periodic assessments of data quality and timeliness, and other factors that can affect the safety of study participants. The board makes recommendations to the PI and NIH on the observed beneficial or adverse effects of the study, interim analysis of efficacy, and other matters. The HNRC’s DSMB meets semi-annually (or more often, as needed) via telephone. We will attempt to add a number of Mexican investigators to this DSMB to ensure that their review is sensitive to cultural considerations. Together, this DSMB will meet by teleconference by Month 2 of the study to review the intervention protocols, assessments and informed consent and to determine whether *a priori* stopping rules and interim analyses should be implemented. The DSMB will also receive all adverse event reports and will review stopping rules and interim analyses, where applicable, semi-annually.

15. POTENTIAL BENEFITS

***CBO Staff***

Benefits to CBO staff from this study will be incidental and in most cases probably minimal. Some staff may derive personal satisfaction from the enhanced knowledge and skills they obtain from their training in recruiting FSWs and delivering the *Mujer Segura* safer-sex intervention, but this will be incidental to their participation in the organizational-level study.

***FSWs***

All participants testing positive for specific STIs (gonorrhea, Chlamydia, syphilis) at baseline and six months post-intervention will receive free treatment on-site (at the MexFam clinic) for these conditions according to Mexico’s Ministry of Health guidelines. Women testing HIV-positive will receive free treatment at the municipal clinic in each participating city. As an incentive to return to the clinic for STI treatment, women will be offered a free health care visit and free medications. In our previous work, free medications have been made available by Mexican pharmaceutical companies.

Other benefits of this research are judged to be minimal to the subjects individually, although some may derive comfort from behavioral changes that they achieve. For example, some women participating in *Mujer Segura* who stopped having unprotected sex with clients at six-month follow-up reported feeling good about themselves and were motivated to encourage and support other FSWs to change their unsafe behavior. Although our intervention is not designed to arrest or abate alcohol or drug use, women will be referred to a social worker at each MexFam clinic for substance use counseling.

16. RISK/BENEFIT RATIO

We judge the importance of the knowledge to be gained from this study to be quite high. First, we expect to significantly increase scientific knowledge of the barriers and facilitating factors associated with implementation of an efficacious behavioral intervention in a high-risk population. Second, implementation of *Mujer Segura* in 12 Mexican cities has the potential for significantly reducing the number of new HIV infections and STIs among FSWs and their clients. This is true both for the participants in the active control condition and for the recipients of the *Mujer Segura* intervention, since in the original *Mujer Segura* study, participants in the active control condition also experienced decreased in frequency of unprotected sex (see Patterson et al., Appendix 1), but due to the absence of a third, “passive” or untreated control arm, it was not possible to determine whether the incidence of HIV or STIs in the active control group declined relative to an untreated group. In view of the minimal risks and the fact that even control group participants are likely to benefit, we hold that the potential benefits predominate.

17. EXPENSE TO SUBJECT

##### Participants will incur no monetary expense by participating in the project. In addition, CBO staff will incur no commitment of their personal time; all the time that they spend providing data to the ethnographers will be during their normal work hours at their respective clinics. FSWs, after being screened and after consenting to enroll in the behavioral intervention study, will spend approximately 1.5 hours at their initial visit (baseline interview, counseling session, and STI testing) and about 45 minutes at their six-month follow-up visit (repeat interview, STI testing).

18. PAYMENT FOR PARTICIPATION

***CBO Staff***

Clinic staff involved in the implementation of *Mujer Segura* who consent to participate in the organizational-level study will receive a $20 honorarium for completing the questionnaire battery, the one-on-one semi-structured interview, and the focus-group meeting at the beginning of their clinic’s involvement in the study. The same amount ($20) will be paid to staff who complete the same set of assessments at follow-up. These amounts will be in addition to the pay that staff receive for being on the job at the time of all these activities. Staff who decline to participate in these activities will be paid as usual for their time on the job but will simply carry out their usual duties. Their working hours will not be curtailed.

***FSWs***

Participants in the *Mujer Segura* behavioral intervention study will receive $30 U.S. for each of two visits (baseline and six-month follow-up). This amount was employed with good success in the trial of *Mujer Segura* that was carried out in several border cities in Mexico between 2003 and 2008 (Project #051182). It is deemed to adequately compensate the participants for potential loss of income without being coercive.

19. PRIVILEGES/CERTIFICATIONS AND LICENSES

**Thomas L. Patterson, Ph.D.** (Principal Investigator) is Professor of Psychiatry at UCSD and has been conducting psychosocial research with populations at risk for HIV infection since 1989. From 2003 to 2008, he was PI of the NIMH-funded *Mujer Segura* behavioral intervention study, which will be more widely implemented in the current study. He will be responsible for overseeing all aspects of the current study.

**Gregory A. Aarons, Ph.D.** (Co-Principal Investigator) is a Research Scientist at Children’s Hospital and Health Center and an Assistant Clinical Professor in Psychiatry at the University of California, San Diego. His expertise is in the area the interface of organizational psychology and mental health services for youth with mental health and substance use disorders. Dr. Aarons’ most recent focus is on the role of leadership in evidence-based practice implementation and the effects of targeted implementation strategies on organizational functioning, service provider functioning, and implementation effectiveness. Dr. Aarons will serve as a co-principal investigator who will provide expert advice on evaluation of provider and organizational factors that potentially influence intervention fidelity.

**Steffanie A. Strathdee, Ph.D.** (Co-Investigator) is a Professor in the Department of Medicine and Associate Dean for Global Health Sciences at UCSD. She is an infectious disease epidemiologist with over 15 years' experience conducting research on marginalized populations, particularly IDUs, who are at high risk of acquiring HIV and viral hepatitis. Together with Dr. Patterson, she will supervise statistical analyses and participate in manuscript preparation.

**Norma Aguirre, M.D.** (Project Manager) is a Mexican-born physician and a Staff Research Associate in the Department of Psychiatry at UCSD. She received her medical training in Mexico. Dr. Aguirre will coordinate and facilitate communication between the San Diego Coordinating Center and MexFam. She will work closely with Mexican researchers and consultants, respond to their problems and concerns, and liaise with administrators at the various CBO sites. Dr. Aguirre will also participate in regular meetings with Drs. Patterson and Strathdee, assist with planning data analyses and writing progress reports and manuscripts for publication.

**Maricela Durá Cobo, M.D.** (Co-Investigator) was appointed Executive Director of MEXFAM in April 2010. Dr. Durá will act as Site Principal Investigator for the 12 MEXFAM clinics, oversee budgets, ensure that recruitment and follow-up goals are met, maintain an IRB to provide initial and continuing review of the protocol and the implemented study, ensure that STI and HIV testing at all the sites is performed according to protocol, act as necessary as a liaison between the implementation sites and the Coordinating Center in San Diego, and submit annual progress reports to the Coordinating Center. She will also assist in the writing of peer-reviewed manuscripts based upon the study data.

**Lawrence A. Palinkas, Ph.D.** (Co-Investigator) is professor of Social Work, Anthropology, and Preventive Medicine at the University of Southern California. He is also Adjunct Professor of Family and Preventive Medicine at the University of California, San Diego. A medical anthropologist, his primary areas of expertise lie within preventive medicine, cross-cultural medicine and health services research. Dr. Palinkas will train, and monitor the qualitative researchers in Ciudad Juarez who will gather ethnographic data at each CBO.

**Shirley J. Semple, Ph.D.** (Co-Investigator) has worked with Dr. Patterson on natural history and behavioral intervention studies for over 17 years, predominantly in HIV-related health risks and outcomes. She was instrumental in developing intervention materials, training intervention personnel, and overseeing day-to-day activities for Dr. Patterson's *Mujer Segura* intervention study. She also served as the U.S. data manager and data analyst for the pilot project. In the proposed study, Dr. Semple will serve as the UCSD onsite investigator. She will provide daily supervision of San Diego project staff, ensure the accuracy of data entry, collaborate on planning analyses, conduct data analyses, assist in writing progress reports, and prepare articles for publication. Dr. Semple will also work closely with the Ciudad Juarez Training Center to ensure that the English versions of intervention materials are continuously updated, and that documentation of changes are recorded.

**Hugo Staines, M.D.** (Co-**Investigator)** is a Professor of Pediatrics and Director of the Biomedical Sciences Instituteat the Autonomous University of Ciudad Juarez (Chihuahua, Mexico). He was responsible for the overall scientific direction and management of the *Mujer Segura* intervention trial in Ciudad Juarez. Dr. Staines will select and monitor counselors who will travel to the Mexican implementation sites in the role of practice experts. He will also select and supervise the project ethnographers who will gather organizational data about the implementing CBOs. Dr. Staines will consult with CBO directors and administrators on training and implentation issues during the study and will work with the investigators at the UCSD Coordinating Center and with Dr. Palinkas at USC to resolve intervention-related problems as they arise. He will also assist in the writing of peer-reviewed manuscripts based upon the study data.

**Mark Chaffin, Ph.D.** (Consultant) is a Professor of Pediatrics and Clinical Associate Professor of Psychiatry and Behavioral Sciences at the University of Oklahoma Health Sciences Center in Oklahoma City, Oklahoma. He currently serves as Director of Research for Developmental and Behavioral Pediatrics and is Co-Director of the National Center on the Sexual Behavior of Youth. He conducts research in areas related to child abuse and neglect, including development and implementation of evidence-based intervention models. Dr. Chaffin will provide advice on the implementation of our train-the-trainer model as well as on working with multiple constituents and stakeholders (e.g., state, county, agency, CBO) to assure the effective implementation of our evidence-based intervention. He will also assist in writing peer-reviewed articles based on the study data.

**Patricia Chamberlain, Ph.D.** (Consultant) is Clinical Director and Senior Research Scientist at the Oregon Social Learning Center. She has conducted several studies on treatment for children, youth, and families in the juvenile justice, mental health, and child welfare systems. Dr. Chamberlain will provide advice on the theory and practice of implementation of evidence-based interventions and be available to help resolve implementation-related problems. She will also assist in writing peer-reviewed articles based on the study data.

**Alejandra Gil Cuervo** (Consultant) is Founder and Director of APROASE, the premier advocacy organization for sex workers' rights in Mexico. APROASE engages government agencies and officials as well as the broader public in Mexico in an effort to reduce stigma and physical violence directed against sex workers, create better working conditions, foster self-esteem and healthier behaviors among sex workers, and to protect their rights. APROASE is a constituent organization of RedTraSex, a network of sex-worker advocacy organizations that covers all of Latin America and the Caribbean. Ms. Gil will provide consultation for the research team and the provider CBOs on establishing relationships of trust with sex worker communities in the various sites, reducing stigma, and overcoming barriers to recruitment. She will also attend community advisory board meetings to ensure that the intervention is appropriately tailored to the needs and culture of Mexican FSWs.

**Helena Chmura Kraemer, Ph.D.** (Consultant) is Professor Emerita of Biostatistics in Psychiatry, Department of Psychiatry and Behavioral Sciences, Stanford University. Dr. Kraemer earned her Ph.D. in Statistics at Stanford University and has focused her career on research issues related to the behavioral aspects of medicine. She was instrumental in the development of the design of this study as well as the analytic plan. She will consult on research conceptualization, research design and power, measurement, data analysis, presentation and interpretation, and all aspects of statistical methodology. She will also assist in writing peer-reviewed articles based on the study data.

**Carlos Magis, M.D., M.P.H.** (Consultant) is the Director of Mexico's National Center for the Prevention and Control of HIV and AIDS (CENSIDA). During the period between 2003 and 2008, he consulted on the design, the cultural adaptation, and the implementation of Dr. Patterson's *Mujer Segura* Study, and he acted as a critical liaison between study staff and the Mexican state and national health authorities. Dr. Magis has co-authored several peer-reviewed articles on *Mujer Segura*. On the current project, he will contribute expertise on issues of HIV/AIDS in Mexico and provide advice to guide the project's development in accordance with the policy and protocols of Mexico's federal government. He will also assist in writing peer-reviewed articles based on the study data.

**Daniela Abramowitz, M.Sc.** (Statistician) is a biostatistician. She will be responsible for converting the survey instrument into a computer program to enable interviews using CAPI. Subsequently, she will maintain the study database, generate follow-up reports, and conduct all statistical analysis for progress reports, abstracts and manuscripts. She will also assist in writing peer-reviewed articles based on the study data.

20. BIBLIOGRAPHY

1. Patterson TL, Mausbach BT, Lozada R, Staines H, Semple, SJ, Fraga M, Orozovich P, Abramovitz D, de la Torre A., Amaro H, Martinez G, Magis-Rodríguez C, Strathdee SA. Efficacy of a brief behavioral intervention to promote condom use among female sex workers in Tijuana and Ciudad Juarez, Mexico. *Am J Public Health* 98 (11): 2051-7, 2008.

2. Rogers EM. *Diffusion of innovations*. 4th ed. New York: The Free Press; 1995.

3. Collins C, Phields ME, Duncan T. An agency capacity model to facilitate implementation of evidence-based behavioral interventions by community-based organizations. *J Public Health Manag Pract.* Jan 2007;Suppl:S16-23.

4. Aarons GA. Transformational and transactional leadership: association with attitudes toward evidence-based practice. *Psychiatr Serv.* Aug 2006;57(8):1162-1169.

5. Aarons GA. Measuring provider attitudes toward evidence-based practice: consideration of organizational context and individual differences. *Child Adolesc Psychiatr Clin N Am.* Apr 2005;14(2):255-271, viii.

21. INDUSTRY STUDIES

Not applicable.

22. FUNDING SUPPORT FOR THIS STUDY

NIH 1 R01 MH087054-01A1. Dates of support: 07/01/2010 – 04/30/2015.
Fiscal Contact: Jennie Sargent, HNRC, Dept. of Psychiatry (jtsargent@ucsd.edu or 619-543-5091).

23. BIOLOGICAL MATERIALS TRANSFER AGREEMENT

Not applicable.

24. INVESTIGATIONAL DRUG FACT SHEET

Not applicable.

25. IMPACT ON NURSING STAFF

There is no involvement of nursing staff in this study.

26. CONFLICT OF INTEREST

Not applicable.

27. CANCER-RELATED STUDIES

Not applicable.

28. PROCEDURES FOR SURROGATE CONSENT AND/OR DECISIONAL CAPACITY ASSESSMENT

No conservatorized persons will be enrolled in this study.

Version date 3-30-2004
